# Poorly Differentiated Neuroendocrine Tumors of the Pancreas: A Comparative Analysis of Primary Versus Secondary Tumors—A Literature Review

**DOI:** 10.3390/biomedicines13061437

**Published:** 2025-06-11

**Authors:** Aleksandr Markov, Akriti Pokhrel, Jen Chin Wang

**Affiliations:** 1 Division of Internal Medicine, Brookdale University Hospital Medical Center, Brooklyn, NY 11201, USA; 2 Division of Hematology and Oncology, Brookdale University Hospital Medical Center, Brooklyn, NY 11201, USA

**Keywords:** neuroendocrine tumors, pancreatic neuroendocrine tumors, poorly differentiated pancreatic neuroendocrine carcinoma, Merkel cell carcinoma, small-cell lung cancer

## Abstract

**Background:** Poorly differentiated neuroendocrine tumors of the pancreas (pd-PNETs) are very rare tumors. Differentiating primary pd-PNET from neuroendocrine carcinomas, which metastasize to the pancreas, can be difficult. We will refer to any neuroendocrine carcinoma with pancreatic metastasis as secondary pd-PNETs. This study evaluates the differences in incidence, clinical picture, outcomes, and treatment between primary and secondary pd-PNETs. **Methods:** A comprehensive search of the pd-PNET database was performed to gather data on incidence, race, age, gender, clinical picture, and outcomes for primary and secondary pd-PNETs. The emphasis was on small-cell lung cancer (SCLC) and Merkel cell carcinoma (MCC) due to their associations with secondary pd-PNET. Additional data from the PubMed database were analyzed, and 12 case reports of primary pd-PNETs were added for clinical characteristic analysis. **Results:** Primary and secondary pd-PNETs exhibit highly similar profiles in terms of age, gender, race, and clinical features. However, treatment strategies are significantly different. Primary pd-PNETs are managed with tumor resection and platinum-based chemotherapy. Primary tumors usually have poor prognosis, with a median survival of 12 months. Treatment for secondary pd-PNETs varies based on the primary tumor. The treatment strategy for metastatic MCC was changed to immune checkpoint inhibitors (ICIs), and survival improved. Tarlatamab also recently showed a good response in the management of SCLC. These findings highlight the need for accurate and timely diagnosis to provide correct treatment. **Conclusions:** Patients with primary and secondary pd-PNETs exhibit similar clinical presentations and epidemiological characteristics. However, when a poorly differentiated neuroendocrine pancreatic mass is identified, it is critical to exclude MCC or small-cell lung carcinoma metastasis, as treatments may be different and prognosis may also be different.

## 1. Introduction

Pancreatic neuroendocrine tumors (PNETs) are rare tumors, with an incidence of one per 100,000 individuals annually, and they account for 1–2% of all pancreatic neoplasms [1]. Well-differentiated pancreatic neuroendocrine tumors (wd-PNETs) are slow growing and have limited proliferative activity; thus, they usually have a good prognosis compared to poorly differentiated tumors [2]. They are usually asymptomatic and can be classified as functional and nonfunctional depending on hormone production [2]. Pancreatic neuroendocrine tumors, which exhibit poor histologic differentiation, can be referred to as poorly differentiated pancreatic neuroendocrine tumors (pd-PNETs). These tumors are always of high grade (G3) and have a KI-67 index >20% by definition [3]. Histologically, pd-PNETs can be classified according to cell type, having either a small cell type or a large cell type [4]. It is very important to distinguish primary poorly differentiated neuroendocrine tumors of the pancreas (pd-PNETs) from pancreatic metastasis originating from other neuroendocrine cancers (NECs), most notably small-cell lung cancer (SCLC) and Merkel cell carcinoma (MCC). In this article, we will refer to pancreatic metastasis from neuroendocrine carcinoma as secondary *pd*-PNETs. This article provides a comparative analysis of primary versus secondary poorly differentiated neuroendocrine tumors of the pancreas. The WHO classification of neuroendocrine neoplasms of the digestive tract is present in Table 1; in our study, we are focusing only on poorly differentiated PNETs.

## 2. Materials and Methods

This study aimed to compare the incidence, clinical picture, treatment, and prognosis of primary pd-PNETs with those of secondary pd-PNETs, given the absence of specific comparative data for poorly differentiated pancreatic neuroendocrine masses of varying origins.

### 2.1. Data Selection

Case reports and case series from the published literature were used in a retrospective study. PubMed and Google Scholar were used as the primary databases to find relevant case reports and case series for primary pd-PNETs and SCLC with pancreatic metastasis and MCC with pancreatic metastasis from 1970 to 2025. The search terms for pd-PNETs included “Poorly differentiated neuroendocrine cancer of the pancreas”, “High grade neuroendocrine carcinoma of the pancreas”, “Gastropancreatic high grade neuroendocrine carcinoma”, “Gastropancreatic neuroendocrine carcinoma”, and “Aggressive Pancreatic Neuroendocrine Tumors”. For primary pd-PNETs, we relied on the epidemiological insights from Gan et al. and the pathological analysis from the case series by Basturk et al. [5,6]. We added clinical data from case reports to address the lack of clinical details in the databases from Gan et al. and Basturk et al. The case reports are summarized in Table 2. For secondary pd-PNETs, we included data for MCC from the database of Pokhrel et al., and for SCLC, we used data from Ugur Gonlugur et al. [7,8].

### 2.2. Inclusion and Exclusion Criteria

We concentrated on poorly differentiated neuroendocrine advanced tumors. Low-grade pancreatic neuroendocrine tumors (G1–G2) and mixed tumors were not included in this study. We did not include case reports and case series not written in the English language.

### 2.3. Data Analysis

To determine incidence, clinical picture, and prognosis, we examined the data from included case reports and case series. Also, the literature on treatment options was reviewed and analyzed. We aimed to compare the clinical presentation of the tumor and analyze the differences in treatment options and prognosis for primary and secondary tumors.

### 2.4. Limitations of This Study

Given the rarity of both primary pd-PNETs and neuroendocrine metastasis in the pancreas, the main limitations of the study were the small sample size used and the retrospective analysis of the case series and case reports.

## 3. Results

### 3.1. Primary Poorly Differentiated Pancreatic Neuroendocrine Cancers

#### 3.1.1. Incidence

Primary *pd-*PNETs are rare and aggressive pancreatic neuroendocrine tumors, characterized by poor differentiation or undifferentiation. Due to their rarity, comprehensive data on their incidence in the United States remains limited. According to research by Ito et al., among all PNETs, 7.5% are usually *pd-*PNETs [21]. As there are limited data for the incidence of *pd-*PNETs in the US, we can approximately estimate the incidence according to a study from the Netherlands Cancer Registry [22]. The incidence of G3 large-cell neuroendocrine carcinoma between 2001 and 2010 was 0.13 per 100,000 persons per year, while G3 small-cell neuroendocrine carcinoma had an incidence of 0.03 per 100,000/year [22], with a total incidence of *pd-*PNETs of 0.16 per 100,000/year.

#### 3.1.2. Age, Sex, and Race

This article utilizes the findings from a database of 485 confirmed pd-PNET cases during the period from 2004 to 2016 [5]. The data were further enriched with clinical findings from the case reports presented in Table 2 [9,10,11,12,13,14,15,16,17,18,19,20]. Among the 485 documented pd-PNET cases, over 61% of patients were older than 60 years old, highlighting a predisposition among the elderly [5]. A small male predominance was observed, as 55% of patients were males [5]. The cohort showed a significant predominance of White individuals, comprising 79% of cases [5]. These findings were also observed in the study conducted by Bastruk et al. revealing a mean patient age of 59 years and a male-to-female ratio of 1.4 [6].

#### 3.1.3. Clinical Picture

Comprehensive data on the clinical presentation of pd-PNET are scarce in existing databases, necessitating reliance on case reports to demonstrate predominant symptoms. A review of the literature from 1970 to 2025 identified 12 relevant case reports, presented in Table 2 [9,10,11,12,13,14,15,16,17,18,19,20].

Abdominal pain was the predominant symptom, reported in 75% (9 of 12) of cases, highlighting its role as a hallmark feature. Weight loss was also frequent, occurring in 66% (8 of 12) of cases. Additional symptoms included nausea, jaundice, pruritus, flank pain, and periumbilical pain. Epigastric pain radiating to the back was observed in some cases, a presentation that may mimic pancreatitis. SCLC metastasis to pancreas has also been reported to present with symptoms of acute pancreatitis, complicating differential diagnosis [8]. Almost all masses were first identified via a CT scan.

#### 3.1.4. Outcome

The prognosis for *pd-*PNETs is generally unfavorable. A study conducted by Basturk, which examined 44 cases, revealed an overall median survival of 12 months [6]. This is similar to the overall survival rate for Gastroenteropancreatic neuroendocrine carcinomas (GEP NECs), which is 11 months, as reported by the NORDIC NEC study [23]. In the study by Basturk et al., 33 patients had a median survival of 11 months and 8 patients had a median follow-up of 19.5 months [6]. In contrast, our case series included patients who survived beyond two years post-diagnosis, highlighting extraordinary responders despite the typical poor prognosis [11,13,16,17,18,20]. This extraordinary response to therapy and long survival is very unusual for pd-PNETs. The median overall survival was 23 months (10.9–35.5 95% CI); however, given the small sample size, it cannot be considered sufficiently statistically significant to draw a conclusion about the outcome.

### 3.2. Secondary Poorly Differentiated Pancreatic Neuroendocrine Cancers

#### 3.2.1. Incidence

A variety of cancers can metastasize to the pancreas. Adsay et al. reported that the incidence of pancreatic metastases was 1.6% in a study of 4955 postmortem cases [24]. The lung was the predominant source, comprising 42% of cases, followed by the gastrointestinal system at 24.7% [24]. Among pulmonary cancers spreading to the pancreas, SCLC accounts for 29% and large-cell lung carcinoma (LCLC) account for 26% [24]. MCC comprises 2.6% of all secondary tumors [24]. Based on the evidence presented, it can be concluded that metastasis to the pancreas from SCLC, LCLC, and MCC represents the most common means of the occurrence of secondary *pd-*PNETs. The incidence rate of MCC in the United States was 0.7 cases per 100,000 person-years in 2013, equating to 2488 cases annually, and this is expected to increase to 3284 cases per year in 2025 due to the aging population [25]. The incidence of SCLC was 4.7 per 100,000/year in 2019 [26].

#### 3.2.2. Age, Sex, and Race

An analysis of SCLC cases from 2000 to 2019 indicated that the median age at diagnosis varied between 60 and 69 years [26]. SCLC historically showed a male predominance: in a study conducted by Gonlugur et al., the male-to-female ratio was 48:22 and the average age was 59 years [8]. The prevalence of SCLC relative to all lung cancer types is consistently greater in the White population than in the Black population [27]. MCC primarily affects elderly patients: in a case series of MCC patients with pancreatic metastasis, most patients aged 60–80 years and the majority of patients were male (68.19%) [7]. Merkel cell carcinoma is identified in 95% of cases among the White population [28].

#### 3.2.3. Clinical Picture

Pancreatic metastases are generally asymptomatic: if symptoms develop, the most common are jaundice and abdominal pain [29]. The tumors in the head of the pancreas can induce obstructive jaundice [8]. Acute pancreatitis due to metastasis is typically caused by the occlusion of the pancreatic duct by metastatic lesions [8]. Though most instances are asymptomatic, pancreatic metastases of Merkel cell cancer (MCC) can appear with abdominal pain and jaundice, sometimes accompanied by nausea, vomiting, or dyspepsia [7].

#### 3.2.4. Outcome

The prognosis for extensive-stage small-cell lung cancer (ES-SCLC) is generally poor. In the study by Gonlugur et al., median survival was 8.25 months for patients without other metastases and only 1.7 months for those with extensive metastatic disease [8]. Most individuals have widespread metastatic disease, and isolated pancreatic metastasis is uncommon [8].

Historically, metastatic Merkel cell carcinoma (MCC) had a poor prognosis with 5-year overall survival (OS) achieved by only 14% of individuals with metastatic disease [30]. According to SEER survival data from 2000 to 2018, the 2-year survival rate was 22.7% and the 5-year survival rate was 17.2% [31]. In the case series of pancreatic metastasis from MCC, the average duration for the diagnosis of pancreatic metastases and subsequent mortality was 6.3 months, ranging from 1 to 24 months [7].

However, novel therapeutic options have significantly changed the prognosis for MCC. The introduction of ICI-based therapy has progressively improved the 2-year relative survival rate for metastatic MCC, rising from 23% (2010–2012) to 54% (2019–2021) [32]. We will further explore treatment options and outcomes for MCC in the Section 3.4.

### 3.3. Differential Diagnosis

Differential diagnosis starts with the evaluation of the clinical presentation. Most patients with primary and secondary *pd-*PNETs are asymptomatic, with incidental findings often driving malignancy workup. When symptoms occur, abdominal pain is the most frequent complaint for both primary and secondary tumors, sometimes accompanied by less common symptoms such as weight loss, nausea, vomiting, jaundice, or epigastric pain radiating to the back. Differential diagnosis is complicated by their similar epidemiological profiles, as patients have similar ages, with male gender and white population being prevalent. Computed tomography (CT) is the main imaging modality for the identification of the tumors [8]. Initially, pancreatic masses may raise suspicion for pancreatic adenocarcinoma, as this type of malignancy is more common [8]. However, when a biopsy shows a neuroendocrine tumor, precise differentiation between primary and secondary *pd-*PNETs is very important as it influences treatment strategies. MCC, SCLC, and primary *pd-*PNETs have different immunohistochemistry characteristics: Merkel cell carcinomas are typically CK20-positive; this is an indicator that distinguishes it from small-cell lung carcinomas [33]. Unlike MCC, metastases of SCLC are CK20-negative but are positive for CK7, neuron-specific enolase, and thyroid transcription factor-1 [33,34]. Consequently, CK20 staining is an important diagnostic step in evaluating *pd-*PNETs to confirm or exclude MCC metastasis.

### 3.4. Treatment

Primary and secondary poorly differentiated neuroendocrine carcinomas of the pancreas have different treatment approaches.

Treatment approaches for primary *pd-*PNETs are different, including combinations of surgery, chemotherapy, and radiation therapy. If patients with primary *pd-*PNETs are good candidates for surgery, they should undergo the procedure [35]. First-line systemic therapy treatment for primary *pd-*PNETs is a combination of Carboplatin/Cisplatin and Etoposide [36]. In our cohort of 12 primary *pd-*PNETs patients, treatment approaches included the following: chemotherapy alone was used in 25%; surgical resection plus chemotherapy was used in 33%; a combination of surgical resection, chemotherapy, and radiation was used in 16%; and palliative/supportive care was used in 16%.

Immune checkpoint inhibitors changed the treatment strategies for MCC. Avelumab was the first ICI that showed an improved and durable response in metastatic MCC for primary management and chemotherapy refractory tumors; the FDA approved avelumab in 2017 [37,38,39,40,41]. Pembrolizumab subsequently showed significantly improved progression-free survival (PFS) and was approved by the FDA in 2018 [42,43,44]. Retifanlimab also showed an effective and durable response [45]. A study by Paulson et al. analyzing 453 patients with metastatic MCC showed significantly improved 2-year relative survival, rising from 23% to 54% [32].

Platinum-based combinations are the standard of care for initial systemic therapy for patients with SCLC [46]. Monoclonal anti-programmed death-ligand 1 (PD-L1) antibodies, such as durvalumab and atezolizumab, have demonstrated improved survival rates when combined with Etoposide and a platinum agent during both induction and maintenance therapy [47,48,49].

Delta-like ligand 3 (DLL3) is highly expressed in SCLC [50]. Tarlatamab, a bispecific T-cell engager, has demonstrated encouraging results in patients who have relapsed on or developed resistance to platinum-based chemotherapy [51,52]. Therapy achieved a 40% response rate, with a median progression-free survival of 4.9 months and an overall survival of 14.3 months [52]. In this extended follow-up, the objective response rate (ORR) was 35.3%, the median duration of the response was 14.9 months, and the median OS was 20.3 months [51]. Tarlatamab has been approved only for ES-SCLC; however, these results give promising options for therapy for other NECs such as primary *pd-*PNETs and MCC. There are several ongoing trials for Tarlatamab use in DLL3 positive tumors, including primary *pd-*PNETs and MCC: NCT04429087 and NCT04471727.

## 4. Discussion

This study provides a comprehensive comparison of primary pd-PNETs and secondary pd-PNETs based on case reports, case series, and retrospective data from 1970 to 2025. Our analysis highlights the rarity, aggressive behavior, and similar clinical presentations of these tumors, which pose significant diagnostic challenges.

Primary and secondary pd-PNETs share similar demographic and clinical profiles: the age of diagnosis is 59–80 years old and male predominance is evident for both tumors. White ethnicity is most prevalent in both primary and secondary pd-PNETs. Both tumor types are often detected incidentally or present with common symptoms like abdominal pain and weight loss. Computed tomography (CT) is the primary modality for detecting pancreatic masses. A comparative summary of primary and secondary pd-PNETs is presented in Table 3.

Outcomes for primary and secondary pd-PNETs have always been poor. However, survival outcomes for secondary pd-PNETs have improved with novel treatment options, highlighting the importance of accurate diagnosis.

The diagnosis of pancreatic neuroendocrine tumors should always include immunohistochemistry. MCC has positive staining for CK20, unlike SCLC and primary pd-PNETs [33,34]. This unique staining profile makes CK20 an essential marker for diagnosing MCC or excluding other tumor forms.

The introduction of ICIs has significantly improved the 2-year relative survival rate for MCC from 23% to 54% [32]. At the same time, Tarlatamab has demonstrated good results in treating metastatic SCLC, achieving a median overall survival of 20.3 months [51]. These therapeutic advancements emphasize the importance of accurately identifying tumor origin, since it guides the selection of the correct treatment and increases the chances for longer survival.

## 5. Conclusions

This study shows the need for differentiating between primary and secondary pd-PNETs, as they require different treatment approaches. Secondary pd-PNETs, especially those originating from MCC, demonstrate a significantly better prognosis with ICI. When a pancreatic tumor has poorly differentiated neuroendocrine histology, distinguishing between primary and secondary pd-PNETs is challenging, as both tumors have similar clinical presentations and epidemiological characteristics. Thus, it is important to confirm or rule out MCC metastasis using CK20, enabling, in time, the initiation of ICI therapy to improve survival outcomes.

## Figures and Tables

**Table 1:** The 2019 World Health Organization classification for neuroendocrine neoplasms of the digestive tract.

	Ki-67 Index (%)	Mitotic Index (HPF^2^/10 HPF)
**Well-Differentiated NEN**		
NET G-1 (low-grade tumor)	<3	<2/10
NET G-2 (intermediate-grade tumor)	3–20	2–20/10
NET G-3 (high-grade tumor)	>20	>20/10
**Poorly Differentiated NEN**		
NEC G-3 (small-cell type and large-cell type)	>20	>20/10
**Mixed neuroendocrine–non-neuroendocrine neoplasm (MiNEN)**		

NEN: neuroendocrine neoplasm; NEC: neuroendocrine carcinoma; HPF: high-power field; NET: neuroendocrine tumor.

**Table 2:** Case reports of primary poorly differentiated pancreatic neuroendocrine tumors (pd-PNETs).

Case	Age	Sex	Metastasis Sites	Clinical Picture	Histology	Diagnosing Method	Diagnosis Confirmation Method	Location	Treatment	Interval Between Disease Establishment and Death
Corrin B. et al. [9]	50	Female	Liver	Fatigue, weakness, weight loss	Small-cell carcinoma	Technetium-99 m	Autopsy	Tail	Palliative/supportive	Not specified
Hobbs R.D. et al. [10]	66	Male	Liver, gallbladder, mesentery, omentum, small and large bowel, adrenals, peritoneum, vertebrae	Weight loss. ascites, jaundice, flank pain, hypercalcemia	Small-cell carcinoma	CT	Autopsy	Head: 8-cm	Palliative/supportive	Not specified
Morant R. et al. [11]	54	Male	Regional lymph nodes, liver, and bone marrow	Abdominal pain radiating to the back, diarrhea and weight loss	Small-cell carcinoma	CT	FNA	NA	Chemotherapy	Alive at 50 months
O’Connor T.P. et al. [12]	62	Male	Direct invasion of the duodenum and anterior abdominal wall	Weight loss, vomiting, fever, symptoms of duodenal obstruction	Small-cell carcinoma	CT	Endoscopic biopsy	Head: 9 cm	Surgery + chemotherapy	2 months
Nakasone et al. [13]	51	Male	Liver and peripancreatic nodules	Abdominal pain, jaundice, pruritus, dark urine, weight loss	NA	CT	FNA	Head 3.4-cm	Chemotherapy + surgical resection	Alive at 2 years
Van Fraeyenhove F. et al. [14]	55	Female	Liver	Abdominal pain, Diarrhea, flushing	Small-cell carcinoma	CT	CT-guided biopsy	Head	Chemotherapy + surgical resection	2.4 months
Yamamoto M. et al. [15]	42	Male	Liver	Abdominal pain and fullness	NA	CT	Tumoral tissue obtained by liver biopsy	Body: 5.6 × 2.5 cm	Chemotherapy	13 months
Kang N.W. [16]	59	Male	Peripancreatic tissue and lymph nodes	Abdominal pain and fullness	Small-cell carcinoma	CT	Pancreatectomy	Body: 3.3 cm	Chemotherapy + ICIs + surgical resection	Alive at 4 years
Tohmatsu et al. [17]	72	Woman	No	Weight loss	Small-cell carcinoma	CT	FNA	Head: 3.2 cm	Chemotherapy + radiotherapy + surgical resection	Alive at 2 years
Fonseca et al. [18]	34	Woman	Liver	Abdominal pain, weight loss, nausea	NA	CT	Biopsy of the liver metastasis	Tail: 5 × 7-cm	Chemotherapy	33 months
Li et al. [19]	63	Male	No	Abdominal pain	Small-cell carcinoma	CT	Exploratory laparotomy.	Head: 6.5 cm × 8.3 cm	Chemotherapy + surgical resection	Alive at 8 months
Elzein et al. [20]	29	Male	No	Epigastric pain radiating to back, nausea, and mild weight loss	Small-cell carcinoma	CT	Biopsy of the perigastric lymph node	Head: 3.7 × 2.9 cm	Chemotherapy + surgery + radiotherapy	Alive at 28 months

**Table 3:** Comparison of primary and secondary poorly differentiated pancreatic neuroendocrine tumors (*pd-*PNETs).

	Primary *pd-*PNETs	Secondary *pd-*PNETs	
		SCLC with Pancreatic Metastasis	MCC with Pancreatic Metastasis
Origin	Pancreas	Lung	Skin
Incidence	≈0.16 per 100.000	Rare	
Age	59 years old in case series study, with the data including 485 patients, the majority of whom were >60 years old	49–69 years	60–80 years
Gender	Male	Male	
Race	White	White	
Predominant Symptoms	Asymptomatic, Abdominal pain, Weight loss	Asymptomatic, Abdominal pain, Weight loss	
Other Symptoms	Nausea, Vomiting, Jaundice, Flank pain, Symptoms of acute pancreatitis	Nausea, Vomiting, Jaundice, Symptoms of acute pancreatitis	
Immunohistochemistry	CK20-negative	CK20-negative	CK20-positive
Treatment	Surgery, radiotherapy, or/and platinum-based chemotherapy	Platinum-based chemotherapy + ICIs (e.g., atezolizumab) Tarlatamab for relapsed/refractory disease	ICIs (e.g., pembrolizumab, avelumab)
Prognosis	Poor prognosis with medial survival of 11–12 months after diagnosis	Better prognosis with therapy, Tarlatamab group achieved medical OS of 20.3 months	Better prognosis with therapy, ICIa increase 2-year relative survival to 54%

## Data Availability

Data are contained within this article.

## Abbreviations

The following abbreviations are used in this manuscript:

pd-PNETs	Poorly differentiated neuroendocrine tumors of pancreas
wd-PNETs	Well-differentiated neuroendocrine tumors of pancreas
MCC	Merkel cell carcnima
SCLC	Small-cell lung cancer
DLL3	Delta-like ligand 3
ICIs	Immune checkpoint inhibitors
GEP NECs	Gastroenteropancreatic neuroendocrine carcinomas
LCLC	Large-cell lung cancer
NEC	Neuroendocrine cancers
